# 

**Published:** 1880-10

**Authors:** 


HERMAN MYNTER, M. D.

CONTENTS:

| | PAGE. |
|---|---|
| **ORIGINAL COMMUNICATIONS:** | |
| State Regulation of the Practice of Medicine, by William D. Granger, M. D., | 97 |
| Induction of Labor in Protracted Pregnancy, by P. W. Van Peyma, M. D., | 104 |
| Sole-Leather Splints—an Improved Process for Making Them, by Bernard Bartow, M. D., | 108 |
| **CLINICAL REPORTS:** | |
| Nephrolithiasis, Nephrotomy, by Herman Mynter, M. D., | 111 |
| A Case of Puerperal Septicemia, by Fred'k Peterson, M. D., | 114 |
| Strangulation of the Small Intestine by the Mesentery of the Ileum, by A. M. Barker, M. D., | 117 |
| **TRANSLATIONS:** | |
| Antiseptics in Ophthalmology, from the Spanish by Lucien Howe, M. D., | 119 |
| **SELECTIONS:** | |
| A Positive Sign of Pregnancy during the First Three Months, | 123 |
| Treatment of Diphtheria, | 125 |
| The Treatment of Consumption, | 128 |
| Abortion through Sympathy, | 130 |
| **SOCIETY REPORTS:** | |
| Buffalo Medical and Surgical Association, | 130 |
| **EDITORIAL:** | |
| The New Medical Law—Another Interpretation, | 135 |
| Sickness of Prof. Julius F. Miner, | 136 |
| Buffalo State Asylum for the Insane—The Opening, | 137 |
| The Buffalo Medical College—Session 1880-81 | 138 |
| **REVIEWS,** | 139 |
| Books and Pamphlets received, | 144 |

